# The London School of Clinical Medicine at the Seamen's Hospital ("Dreadnought"), Greenwich

**Published:** 1907-09-07

**Authors:** 


### THE LONDON SCHOOL OF CLINICAL MEDICINE AT THE SEAMEN'S HOSPITAL ("DREADNOUGHT"), GREENWICH.

Of the various post-graduate schools that have been established in London, the last to come upon the field is the London School of Clinical Medicine at the *Dreadnought* Hospital, Greenwich. Though many facilities have been provided for post-graduate study in London within recent years, this country is still, as compared with some other centres in Europe and America, behindhand in the opportunities it offers to medical men to return to their own or other hospitals from time to time to burnish the armour of their technical knowledge and to make themselves familiar with the latest advances in medical science. So far as London is concerned, it is possible to attend classes and cliniques under the auspices of various colleges and associations, many of which are carrying on most excellent work. The field, however, is much larger than all the established post-graduate institutions can undertake, and so it came about that when Mr. Johnson Smith retired from the service of the Seamen's Hospital Society at the beginning of last year, the Board of Governors of that national and excellent charity resolved to take advantage of the occasion to establish there a school of medicine for the convenience of qualified men. The Board were prompted to embark upon this scheme by the fact that the basis for a clinical school was already laid at the *Dreadnought*, where Mr. Johnson Smith had, over a long series of years, successfully conducted classes in operative surgery, and by the assurance of the medical staff that the clinical material available within the walls of the hospital was not only abundant, but, in many respects, unique. Further, it was felt that the success that attended the foundation of the Tropical School at the branch hospital augured well for the future of a Clinical School at the parent Institution: a school that would be a supplemental extension of the teaching facilities already afforded in tropical diseases and that would enable students of tropical medicine to extend the area of their studies in medicine and surgery, and would also throw open to qualified men generally the valuable clinical resources under the Board's control.

This wise and enlightened policy is much to be commended as being in the best interests of the patients for whom the Seamen's Hospitals are provided, and at the same time of immense potential value to medical science and the mitigation of human suffering.

### The Teaching Staff.

In order that this new school should be established on a broad and influential basis, the organising committee decided that it was requisite as a first step towards ultimate success, that the staff should be augmented by the addition—first of seniors who should be men of established reputation; and, secondly, of juniors who should undertake the work of an out-patient department, hitherto undeveloped at the *Dreadnought* Hospital. Steps were taken to ascertain how far these requirements could be fulfilled, and it was speedily seen that the scheme found widespread favour with leading members of the profession. A glance at the names of those who have associated themselves with the movement by becoming members of the senior teaching staff will be sufficient to indicate that the movement has met with gratifying recognition among those who represent the highest and most distinguished interests both in medicine and surgery.

The appointments to the junior staff have been no less gratifying, and the names of those already appointed conclusively show that a skilful selection has been made and a powerful body of active and enthusiastic younger men brought together to carry on the work of the junior offices.

In addition to this, there has been appointed a large staff of extra-mural teachers who will carry on the work of special departments in subjects which are outside the scope of the work which the hospitals of the Seamen's Society are established and maintained to perform. These hospitals are founded for the succour of seamen, and, therefore, with few exceptions, male patients only are admitted to their wards. But in order that the curriculum of the London School of Clinical Medicine may be comprehensive enough to include all branches of practical instruction, arrangements have been made for the affiliation with the Seamen's Hospital of the Royal Waterloo Hospital for Children and Women, the Bethlem Hospital for Mental Diseases, and the York Road Lying-in Hospital. These hospitals are all situated on the south side of the Thames and are linked to one another by tram and rail, so that post-graduates attending the services of the new school are enabled to take out courses of instruction in every department of medicine and surgery. This is an admirable arrangement, which from the point of view of comprehensiveness nearly approaches the great polyclinics of America and the Continent.

### The Clinical School.

The Clinical School caters for qualified men only, because it recognises that the admixture of graduates with undergraduates for teaching purposes is inexpedient and unprofitable; it is prepared to arrange classes to meet the requirements both as to time and subjects of those who desire to enter upon a course of study under its auspices. The fees are assessed upon a scale that bring its offices within the reach of all who desire to avail themselves of the exceptional educational advantages which it offers. The certificates of the school are recognised by the Admiralty, the War Office, the Colonial Office, the University of London, and other educational bodies.

### The "Dreadnought" Hospital.

The *Dreadnought* Hospital, which is the centre of the organisation, is situated at Greenwich, close to both railway stations, and within 30 to 40 minutes' reach of London by rail or tram. The hospital contains 250 beds, and these are continuously occupied by cases of wide and varying clinical interest. The admissions to the *Dreadnought* Hospital differ from those at most other hospitals in that cases of tuberculosis and venereal disorder are admitted. The tuberculosis cases are segregated on one of the upper floors, where between 30 and 40 beds are reserved for their use. The floor is in direct communication with the roof of the building, which is flat and is utilised for treatment in the open air. It is in contemplation to segregate the venereal cases in a similar way, but at present neither the accommodation in the hospital nor the funds at the disposal of the Board of Management permit of this being fully accomplished.

The beds are about equally divided between medical and surgical cases, and in them may be seen every variety of disease. On one floor in a medical wing we saw, in the course of a recent visit, amidst many cases of interest, patients suffering from Bright's disease, leprosy, locomotor-ataxia, beri-beri, pneumonia, dysentery, aortic disease, aneurysm, neurasthenia, and malignant disease of the stomach. This indicates fairly accurately the class and extent of " material " met with throughout the hospital on both the surgical and medical sides. The wards are mostly small, many of them containing no more than three beds: there are, therefore, unusual opportunities for the individual examination of cases.

### Out-patient Work.

The out-patient department has been re-organised and equipped with the latest modern requirements. Besides the ordinary medical and surgical consulting rooms, it provides special departments for diseases of the eye, diseases of the skin, and diseases of the ear, throat, and nose. There is a large and admirably-arranged operating theatre for in-patients, and a smaller one in the out-patient department. There are also two laboratories; an extensive and rapidly-growing pathological museum, and convenient post-mortem rooms, where pathology and operative surgery are taught. In the pathological department arrangements have been made whereby investigations of all sorts—chemical, microscopical, biological, etc.—are undertaken at moderate fees. Morbid specimens may be sent by any practitioner, and a careful report may be depended upon. The *x*-ray room is fitted up with the most modern apparatus and is kept continually busy. For the purposes of the school there are lecture rooms and a library; while the comfort of those who attend the classes has been kept in view by the provision of comfortable reading and smoking rooms. Men engaged in practice in the neighbourhood of the hospital have been invited to join the school as associates at a nominal fee, and a considerable number have already availed themselves of the privilege. By this plan it is possible for those within reach to visit the hospital and attend the cliniques when it is convenient for them to do so—a most excellent arrangement for busy men.

### Lodgings.

Within easy access of the hospital, either in Greenwich, Blackheath, Lewisham, or Westcombe Park, there is an abundance of comfortable rooms to be obtained, the addresses of which are inscribed in a register at the hospital, so that those who prefer to live near their work rather than in town, can readily find a choice of suitable accommodation. The scheme of work at the Clinical School is comprehensive and well arranged. Cliniques in medicine, surgery, and special departments are held between two and five o'clock every afternoon; while the work of the out-patient department is carried out during the morning. Special classes are arranged on various subjects in accordance with the demand and at hours to suit the majority of those who go to make them up. From the sketch thus given, it will be seen that those who come to this school have a wide field from which to choose the subjects which they may specially desire to study, besides having the whole practice of the *Dreadnought* and of the affiliated hospitals thrown open to them for bedside investigation and research.

We are glad to know that a large measure of success has already attended the work at this School. It is still in its infancy, but it cannot fail to attract a yearly increasing number of post-graduate students and to become an important factor in the life of the medical teaching schools of London. We are given to understand that any medical man, on presentation of his visiting card, is made welcome at the hospital and is cordially invited to take part in the cliniques of the day.

